# Epidemiology, Clinical Manifestations, and Long-Term Outcomes of a Major Outbreak of Chikungunya in a Hamlet in Sri Lanka, in 2007: A Longitudinal Cohort Study

**DOI:** 10.1155/2012/639178

**Published:** 2012-02-01

**Authors:** Senanayake A. M. Kularatne, Sajitha C. Weerasinghe, Champika Gihan, Sujantha Wickramasinghe, Samath Dharmarathne, Asanka Abeyrathna, Thilak Jayalath

**Affiliations:** ^1^Department of Medicine, Faculty of Medicine, University of Peradeniya, Peradeniya, 20400, Sri Lanka; ^2^Department of Community Medicine, Faculty of Medicine, University of Peradeniya, Peradeniya, 20400, Sri Lanka

## Abstract

Chikungunya outbreaks occurred in the central province, Sri Lanka in 2006. This community-based study reports the epidemiology and the natural history of the infection from an affected village. Of the 199 families and 1001 individuals in the village, 159 (80%) and 513 (51%) were affected, respectively, comprising 237 (46%) males with peak incidence at 40–50 years. The acute illness caused polyarthritis in 233 (46%), and of them 230 (98%) progressed to chronic arthritic disability (CAD). Of the CAD patients, 102 (44%) had recovered in 141 days (range 30–210 days) from the disability state whilst 128 (56%) had persisting disability which lasted 12, 24, and 36 months in 41 (17.8%), 22 (9.5%), and 14 (6.1%) individuals, respectively. Carpal tunnel syndrome (CTS) manifested in 110 (21%). Females showed preponderance for complications over males: acute arthritis 147 versus 86, *P* = 0.001; CAD 136 versus 84, *P* = 0.029; CTS 88 versus 22, *P* = 0.001; relapses 105 versus 68, *P* = 0.001. Chikungunya was highly communicable and caused lasting crippling complications.

## 1. Introduction

Chikungunya is a crippling mosquito-borne disease caused by the chikungunya virus that has recently emerged as a significant public health problem in Sri Lanka [1, 2]. In February 2005, chikungunya infection hit islands of the southern Indian Ocean affecting up to 200,000 inhabitants and spread to India in 2006 resulting in 1.38 million symptomatic illnesses in Central and southern India [3–8]. The worst affected was Reunion Island where 35% of its inhabitants were affected by the epidemic [9]. First isolated in Tanzania in 1953, chikungunya virus belongs to the family *Togaviridae*, genus *Alphavirus* with three genotypes: East African, West African, and Asian [5, 10]. A study showed that the recent epidemic in South-East Asia has been due to five genetically distinct subpopulations of chikungunya virus strains belonging to East, Central, and South African (ECSA) lineage and evolutionarily more related to Indian strain [8].

Chikungunya is a zoonotic disease, and the life cycle involves primates and *Aedes* mosquitoes [11]. There is no effective vaccine to prevent the disease, so that mosquito control is the appropriate strategy to control and to contain the infection [12]. The acute disease is self- limiting, and only symptomatic treatment is necessary. However, the chronic phase is crippling for varying time periods. A few studies have examined the chronic phase of the illness, but the overall picture and natural history were not well documented [9, 10, 13, 14]. Thus, studying a cohort of patients from an affected community with close followup for a long period would certainly elucidate more information about the infection.

The disease struck Sri Lanka in mid October 2006 affecting many parts of the Island, and there were isolated pockets of high incidence of infection [2, 15]. One such area was Galagedara-Madige, a village in Kandy District, Sri Lanka, which was the focus of our study. In this village a substantial number of inhabitants were affected by the epidemic, and large numbers have been suffering from its chronic complications even six months after the infection. The objectives of the study were to describe the epidemiology, clinical picture, complications, outcome, and natural history of the chikungunya infection among all households of the village.

## 2. Methods

### 2.1. Subject Enrolment

We carried out a household survey in Galagedara-Madige, the index village of the study situated in Kandy District, Sri Lanka, during a period of three weeks from May to June 2007. At that time seven months had elapsed from the onset of the epidemic in the village. The village has been exclusively inhabited by Sri Lankan Muslims with high population density. The households of surrounding villages of Galagedara-Madige were also interviewed for comparison of results. 

Ethical clearance was obtained from the Ethical Committee, Faculty of Medicine, University of Peradeniya, Sri Lanka. Permission to interview people in the village was sought from the local health authorities, Deputy Director of Health Services, and Medical Officer of Health-Galagedara.

### 2.2. Data Collection

Interviewers were three qualified medical graduates who first underwent familiarization with the data collection sheet and the format of interview. A structured, interviewer-completed questionnaire was used for the data collection. In order to avoid interobserver variation, a common format was agreed upon after deliberations amongst the team members. 

The questionnaire consisted of two parts namely “family data sheet” and “individual data sheet.” Part one, “family data sheet” was used to collect the demographic data (name, age, gender, contact details, and number of family members) of the family and part two, “individual data sheet” was used to compile data with respect to each patient who presently suffers from chikungunya or had a previous attack. Part two of the questionnaire contained details such as onset of the disease, clinical features, disability state, nonarthritic complications, and past medical history. Where available, the patient's clinical notes were inspected to gather more details. Individuals with chronic arthritic disability were followed up periodically in the field at 6, 12, 24, and 36 months till June 2010.

### 2.3. Diagnosis of Cases and Disability

Though viral diagnostic tests are used for the confirmation of the infection, its availability in an outbreak is limited [5]. Hence, reliance on clinical data and circumstantial evidence was stressed. While interviewing the individuals, diagnosis of cases was made clinically, considering the epidemiological and circumstantial evidence. Clinical, laboratory, and serology records were also examined for the confirmation of the diagnosis, since local doctors had documented the diagnosis during the epidemic. Validation of clinical diagnosis was made based on published data from Kandy district during the same periods [16]. Diagnosis of chronic arthritic disability was made on clinical grounds if the patient was having or had arthritic disability for more than 14 days after the defervescence of the acute illness.

### 2.4. Statistical Analysis

All the data collected were coded and computerized using Microsoft Excel Software (Microsoft Office 2007 Package). Analysis of the data was done using the statistical package, SPSS version 17.0 for Windows.

Calculation of frequencies and percentages was the first step in the analysis of the demographic data. The associations between rates of chikungunya infection and the demographic data were then analyzed using the Chi-square test and “Student's” *t*-test. The correlation coefficients between the number of patients per family and the number of individuals in the family were also calculated.

The Chi-square test was used to analyze the association between demographic data and acute illness, disability, carpal tunnel syndrome, and relapses.

## 3. Results

### 3.1. Basic Epidemiological Analysis

**Figure 1:**
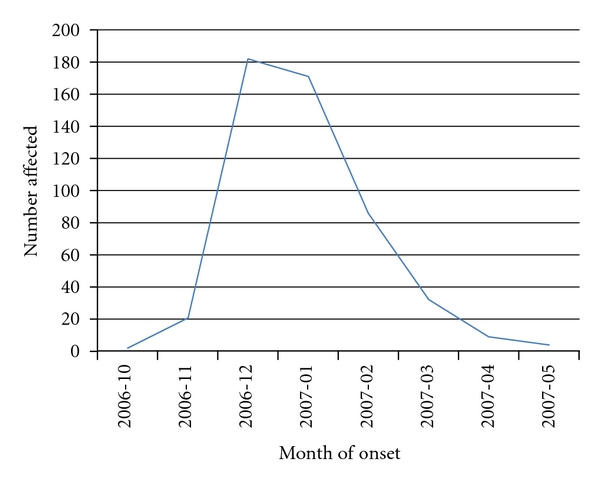
Temporal relationship of chikungunya incidence in Madige.

Of the 1832 individuals interviewed in the study, 1001 (55%) were from Galagedara-Madige (study village), and the rest 831 (45%) were from the neighboring villages of Galagedara-Madige (Control group). Of the 1001 individuals of Madige, 513 (51%) were infected with chikungunya compared to 47 (6%) cases in the control group (*P* = 0.000)  during the outbreak of chikungunya in the region from October 2006 to May 2007. The incidence of cases had peaked during December 2006 to January 2007 in the study village Madige (Figure 1). 

**Figure 2:**
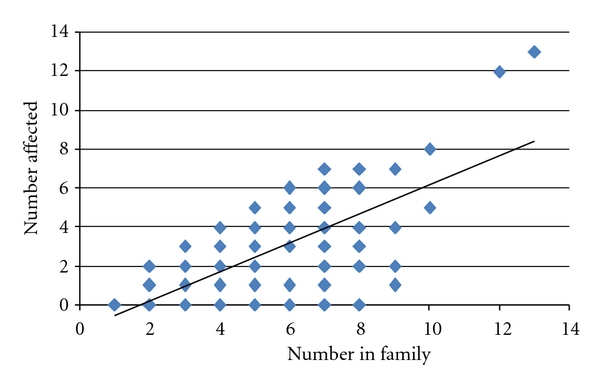
Relationship between numbers of affected per family and number of individuals per family.

There were 199 families living in Madige, and of them 159 (80%) families were affected during the epidemic. There was a positive correlation between the numbers of individuals affected per family and the total number of family members. (Pearson's correlation coefficient = 0.497, *P* = 0.01) (Figure 2). 

The study population of Madige (1001) comprised 485 (49%) males and 516 (52%) females (Table 1). 

Of the affected 513 (51%) people, 237 (46%) were males and 276 (54%) were females (*P* = 0.144) whilst 160 (31%) were house wives, 120 (23%) were school students, and 100 (20%) were businessmen. The incidence of the disease had increased with increasing age and peaked at 40–50 years with the lowest incidence in children as shown in Table 2. The mean age of the affected group and the nonaffected group was 35 years (range 1–90) and 26 years (range 1–98) (*P* = 0.000), respectively. 

### 3.2. Analysis of Acute Illness

Of the triad of clinical features (fever, rash, and joint disease) used for the diagnosis of chikungunya infection, 100 (20%) had the rash, 233 (46%) had polyarthritis, and 430 (85%) had arthralgia. Of the patients with the entire triad (61), the majority were females (*P* = 0.002), but the incidence of arthralgia did not show a significant gender-based difference (*P* = 0.56) (Table 1).

Among the chikungunya affected 513, there was a single death, and details of the postmortem could not be traced. Of the remaining 512, 233 (46%) had polyarthritis comprising 86 (37%) males and 147 (54%) females (*P* = 0.000). Of them, 107 (46%) had arthritis along with fever while 114 (49%) had arthritis at the defervescence, and 12 (5%) had arthritis before the onset of fever. 

Involvement of joints during the acute phase showed more involvement of weight- bearing joints: ankle 173 (74%), knee joint 96 (41%), and feet 75 (32%) than other joints: wrist 34 (14%) and back 7 (3%). 

### 3.3. Analysis of Chronic Arthritic Disability

Of the 512 chikungunya-affected group, 230 (45%) had chronic arthritic disability (CAD) that comprised 99% of patients who had initial acute polyarthritis with female preponderance (*P* = 0.029) (Table 1). Distribution of the joint involvement was the same as in acute arthritis with persistent disability in the weight-bearing joints. The proportion with CAD had increased with advancing age as shown in Table 3. 

Considering the first two age categories, the proportion who had CAD was 10% and 27%, respectively, and this difference was significant (*P* = 0.005). The mean ages for the CAD group and the nondisability group were 44 years (range 2–90 ) and 27 years (range 1–90) (*P* = 0.000), respectively. Waxing and waning of chronic arthritis was reported by 204 (89%) patients, and the commonest cause of relapses was work-related activity. 

Of the 230 who complained of CAD 102 (44%) had recovered from the disability state (recovered group 20% of 512 patients with chikungunya) whilst 128 (56%) had persisting disability (persistent group) at the time of the interview. The mean duration of the disability in the recovered group was 141 days (range 30–210 days). The severity of arthritic disability was graded as pain, pain with swelling, limitation of movements, limitation of function, and bed ridden. Out of these five categories, pain in the joints was the commonest CAD, and it was found in 59% followed by limitation of function (31%) of the patients. The outcome of the persistent group will be assessed periodically over next three years. 

Of the 512 with chikungunya, 34 (7%) patients gave a past history of arthritic condition. Out of them 21 (64%) had an exacerbation of arthritis with chikungunya infection, and 12 (36%) patients remained quiescent (*P* = 0.037). Also 18 (53%) of them had disability, and 16 (47%) did not have disability (*P* = 0.454).

The carpal tunnel syndrome (CTS) was the commonest nonarthritic complication following the chikungunya infection (Table 4). 

**Figure 3:**
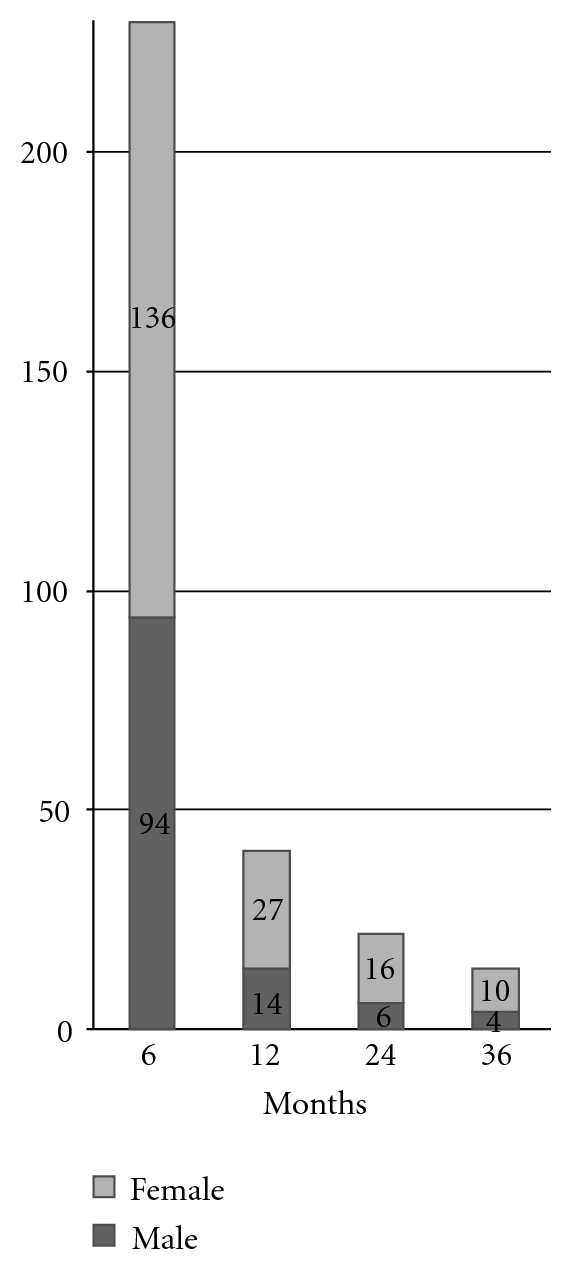
Number of individuals with chronic arthritic disability (CAD) at followup.

The proportion with CTS had increased with the age. In the age group of 0–12 years, there were no reported cases of CTS whilst the incidence increased with age: in 13–22-year group 5%, 33–42-year group 31%, 43–52-year group 37%, and 53–62-year group 36%. Furthermore, the incidence of CTS had significantly increased in females, in patients with past history of arthritis and in the chronic arthritic disability group. Subjects with CAD were followed up at 6, 12, 24, and 36 months following the acute clinical illness. The numbers with the CAD are as follows: 230 (100%), 41 (17.8%), 22 (9.5%), and 14 (6.1%), respectively (Figure 3).

## 4. Discussion

This survey describes the pattern of spread, prevalence of long-term sequels, and the natural history of chikungunya infection which swept across a hamlet in central Sri Lanka during the recent outbreak of the disease. This village is a compact mass of houses which is inhabited only by the Sri Lankan Muslim community who prefers to live as close extended family, and this fact would have contributed to the higher incidence of the disease compared to the neighboring villages. The peak of the infection lasted two months bringing the life in the whole village to a standstill with economic difficulties. We found that the infection had affected more adults than children and a concurrent sharp rise of the incidence within households of big families. Hence, the alarming rapidity of spread of the infection is an epidemiological feature to be investigated. 

Morbidity was more among females as they outnumbered the males in acute polyarthritis, chronic arthritic disability (CAD), carpal tunnel syndrome (CTS), and relapses of arthritis (Table 1). 

Polyarthralgia was the dominant symptom in the acute stage of the illness and only about 20% gave a history of a skin rash. Acute polyarthritis affected 45% of the cohort, and of them 50% of patients had developed arthritis with the defervescence. Furthermore, weight-bearing joints were more affected. Interestingly, 99% of patients with acute polyarthritis had progressed to CAD which was characterized by waxing and waning in severity and very often exacerbated by physical activities (Table 4). The acute arthritis and CAD were much less in the paediatric age group (*P* = 0.001), and what made them less susceptible to these complications is an open-ended question. The duration of the CAD varied from 30 to 210 days in the recovered group accounting for 44% of CAD, but the balance 56%, classified as persistent, group would probably continue with symptoms for hitherto unknown period. At the end of three years of followup of CAD, we found 6.1% were still suffering with debilities. 

The reason why the study population had significant arthritic complications is an unanswered question, and it needs further research, focusing on genetic studies or HLA studies to identify any predisposition. The CTS was the commonest nonarthritic complication (22%) in our study, and this was commoner in females (*P* = 0.001), and a smaller proportion of patients had symptoms suggestive of a postviral fatigue syndrome. 

Chikungunya virus is not a stranger to Sri Lanka as the first epidemic was reported in the early 1960s, followed by quiescence until the current epidemic which started in mid October 2006 [2, 17]. There were more than 37, 000 cases reported in first few months from different parts of the island, but this was a tip of the iceberg due to underreporting [2]. Devastating outbreaks occurred in pockets of close communities, and one such a hotspot was the index village of our study. Viral transmission from human to human through direct contact with highly viremic blood was postulated in France [4]. 

The disease is transmitted by *Aedes* mosquito, which is the vector of Dengue fever and chikungunya, found in Sri Lanka [16]. It is an urban mosquito and breeds in clear water. However, overcrowding of houses would have led to poor hygiene and prevalence of mosquito breeding sites. A study done in India showed that high population density, lack of adequate vector control, and poor hygiene were important risk factors in the population [18].

In the acute illness, we recorded skin manifestation in 20% of cases, but other studies had observed 40–50% involvement of skin [6, 14]. More data are available on arthritis that had affected 73–80% patients in some published series and had chronically persisted 4 months and 3 years in 33% and 10% of patients, respectively [6, 10, 14]. Furthermore, in a series of 47 travelers returning to France, 38 (81%) had chronic peripheral rheumatism affecting mainly ankle, wrist, fingers, and toes after the 10th day of illness contrary to the findings of our series where 44% of patients had chronic arthritic debility mainly affecting weight-bearing joints [9]. Further, the same study showed a preponderance of arthritis to previously injured joints, similar to our observation where there was a significant association between past arthritis and the CAD [9]. Among the nonarthritic complications, CTS was the commonest complication (*n* = 110, 22%). A previous study showed that 10 out of 47 patients had CTS [9], but further analysis of the CTS has not been done. We found that the incidence of CTS was more associated with females, chikungunya arthritis, past medical history of arthritic conditions (Table 4), and age. 

Interestingly the outbreak of chikungunya in 1965 had reported less joint disease and nonarthritic complications than the current series [17]. Furthermore, reemerged infection appeared to be more severe than the past disease [10]. Plausible explanations for the increased morbidity of the current infection include mutations of the virus, absence of herd immunity, lack of vector control, and globalization of travel and trade [6, 10]. Sri Lanka records high incidence of chikungunya among the regions during the ongoing epidemic in the Indian Ocean. A large number was affected during the epidemic, and they are still suffering from its chronic complications. This has affected the activities of daily living and the well-being of the substantial number of people in the community. Unfortunately, there is no evidence based-treatment policy for the management of CAD except for its natural healing and alleviation of symptoms. Hence, proper understanding of the pathophysiology of chronic disability and development of an effective treatment regimen would be an urgent task.

## Figures and Tables

**Table 1:** Gender-based analysis of features.

Variable [*N*]		Male %	Female %	*P* Value^a^
Population of Madige (1001)		485 (48)	516 (52)	
Affected people in Madige (513)		237 (46)	276 (54)	0.144
Triad of symptoms (61)		17 (28)	44 (72)	0.002
Arthralgia (430)		191 (44)	239 (56)	0.560
Arthritis (233)		86 (37)	147 (63)	0.000
Chronic arthritic disability (230)		94 (41)	136 (59)	0.029
Carpal tunnel syndrome				
Relapses	Present	22	88	0.000
	Absent	214	184	
			
	Present	68	105	0.000
	Absent	166	167	

^a^ *χ* ^2^ statistics.

**Table 2:** Incidence of chikungunya infection by age group.

Categorized age	Chikungunya fever [ *N*, % ]*				Total
	Infected		Noninfected		
0–12	93	38%	152	62%	245
13–22	74	45%	91	55%	165
23–32	81	51%	78	49%	159
33–42	80	58%	57	42%	137
43–52	76	62%	47	38%	123
53–62	54	59%	38	41%	92
63–72	41	71%	17	29%	58
73–82	12	67%	6	33%	18
83–92	2	67%	1	33%	3
93–102	0		1	100.00%	1

**Total**	**513**	**51%**	**488**	**49%**	**1001**

*Count, % within categorized age.

**Table 3:** Distribution of chronic arthritic disability in age groups.

Age category	With disability [*N*, %]		Without disability [*N*, %]		Total
0–12	9	9.9	82	90.1	91
13–22	20	27.0	54	73.0	74
23–32	35	43.2	46	56.8	81
33–42	44	55.7	35	44.3	79
43–52	46	60.5	30	39.5	76
53–62	36	67.9	17	32.1	53
63–72	31	75.6	10	24.4	41
73–82	8	66.7	4	33.3	12
83–92	1	50.0	1	50.0	2

**Table 4:** Nonarthritic complications among chikungunya-affected patients.

Complication	Number affected [*N*, %]	
Carpal tunnel syndrome	110	21.5
Postviral fatigue syndrome	8	1.6
Thrombophlebitis	2	0.4
Respiratory tract infections	4	0.8
Calf swelling	1	0.2
Facial swelling	3	0.6
